# Multimodal biomarkers for early detection and immunotherapy assessment in hepatocellular carcinoma: integrating liquid biopsy, AI-based imaging and tumor immune profiling

**DOI:** 10.3389/fgene.2026.1951687

**Published:** 2026-10-05

**Authors:** Yuli Zhao, Xiaolei Chen, Hanyu Zhao

**Affiliations:** 1 School of Food & Pharmaceutical Science and Technology, Guangzhou College of Technology and Business, Guangzhou, China; 2 Department of Medical Oncology, The First Affiliated Hospital of Henan University of Science and Technology, Luoyang, China

**Keywords:** cell-free DNA, deep learning, hepatocellular carcinoma, immunotherapy, liquid biopsy, radiomics

## Abstract

Hepatocellular carcinoma (HCC) commonly develops in chronically diseased liver and remains challenging because of late detection, heterogeneity, recurrence and limited hepatic reserve. Immune checkpoint inhibitor (ICI)-based combinations improve outcomes in advanced HCC, but durable responses occur in only a subset of patients. This structured narrative review evaluates biomarkers by intended use: surveillance and early detection, pretreatment prognostic stratification, treatment selection, on-treatment monitoring, neoadjuvant response assessment and postoperative minimal residual disease (MRD) detection. Serum alpha-fetoprotein and clinical scores are accessible but largely reflect tumor burden, inflammation or liver function. Cell-free DNA methylation and fragmentomics provide early-detection and tissue-of-origin signals, while circulating tumor DNA tracks molecular burden and clonal evolution. Tissue and spatial profiling clarify immune responsiveness and resistance, whereas radiomics and deep learning noninvasively assess whole-tumor and peritumoral heterogeneity. These modalities are complementary but require distinct evidence standards. Most early-detection studies use case-control or enriched cohorts, and most immunotherapy associations are retrospective, prognostic or monitoring-related. No biomarker yet supports routine selection among ICI regimens. Unlike modality-specific summaries, this review aligns each biomarker with a defined clinical decision and evidentiary category and proposes a longitudinal framework integrating baseline blood, imaging and tissue features with on-treatment dynamics, preoperative reassessment and postoperative molecular surveillance. Clinical translation requires standardized assays, representative multicenter cohorts, locked external validation and biomarker-guided trials demonstrating value beyond established criteria.

## Introduction

Hepatocellular carcinoma (HCC) is the predominant primary liver malignancy and usually develops in the setting of chronic liver disease, including hepatitis B virus infection, hepatitis C virus infection, alcohol-associated liver disease and metabolic dysfunction-associated steatotic liver disease (MASLD) (Singal et al., 2023). The contribution of MASLD is increasing in many populations (Y et al., 2024). Despite advances in antiviral therapy, surveillance, locoregional treatment, systemic therapy and surgery, delayed diagnosis, molecular heterogeneity, frequent recurrence and compromised hepatic reserve continue to limit long-term outcomes (Singal et al., 2024).

In clinical practice, treatment decisions still depend largely on tumor burden, liver function, performance status and radiological stage. These variables are indispensable, but they do not fully capture the biological diversity that drives tumor aggressiveness, immune escape, treatment resistance and recurrence (Foy et al., 2023). Patients with similar clinicoradiological characteristics can therefore have markedly different responses and outcomes, a limitation that has become increasingly apparent in the era of immunotherapy.

ICI-based regimens, particularly combinations with anti-angiogenic agents or other immune therapies, have improved outcomes in advanced HCC. Nevertheless, objective responses remain limited to a minority of patients: the RECIST 1.1 objective response rate was 27.3% with atezolizumab plus bevacizumab in IMbrave150% and 20.1% with the STRIDE regimen in HIMALAYA (Rimassa et al., 2023; Rimassa et al., 2025; Sangro et al., 2024). These trials involved different populations and should not be interpreted as direct cross-trial comparisons. The remaining variability reflects the complex interplay between tumor biology, immune context, liver function and treatment exposure (Rimassa et al., 2023). Candidate biological correlates include PD-L1 expression, tumor mutational burden, WNT/β-catenin activation, immune-cell states, circulating markers and imaging-derived signatures, but none is sufficiently validated for routine selection among ICI regimens.

ICI-based regimens, particularly combinations with anti-angiogenic agents or other immune therapies, have improved outcomes in advanced HCC. However, responses remain highly variable across patients, reflecting the complex interplay between tumor biology and the immune microenvironment. (Gordan et al., 2023). This clinical heterogeneity highlights the urgent need for robust biomarkers capable of guiding patient selection, predicting therapeutic benefit and enabling longitudinal disease monitoring.

Systemic immunotherapy is also being investigated in earlier-stage HCC. Upfront resection remains the standard curative approach for patients who are suitable surgical candidates; neoadjuvant systemic therapy remains investigational (Zhang Y. et al., 2025; Cicerone et al., 2025). It may induce tumor regression, treat occult micrometastatic disease and prime antitumor immunity while tumor antigens remain present, but it can also cause toxicity, delay surgery or permit progression beyond resectability (Ho et al., 2021; Li M. et al., 2024; Griffiths et al., 2022). Biomarker studies in this setting should therefore address a specific decision: candidate selection, early continuation, surgical timing, pathological response or postoperative residual disease detection.

These challenges have stimulated interest in noninvasive biomarkers that integrate molecular profiling with quantitative imaging. Cell-free DNA (cfDNA) methylation and fragmentomics may support HCC detection and risk assessment, whereas circulating tumor DNA (ctDNA) can reveal tumor-derived molecular alterations and clonal evolution (Sivapalan et al., 2023; Tran et al., 2021). Radiomics and deep learning can convert routine CT and MRI into quantitative representations of tumor phenotype, vascular architecture, spatial heterogeneity and putative immune contexture (Song et al., 2024). This Review examines how serum, liquid-biopsy, imaging and tissue biomarkers can be aligned with defined clinical questions across the HCC care continuum, with particular emphasis on immunotherapy and neoadjuvant assessment.

### Literature search and evidence selection for this narrative review

This article was designed as a structured narrative review rather than a systematic review or meta-analysis. PubMed/MEDLINE was searched for English-language, peer-reviewed publications from 1 January 2016 through 31 August 2026. Preprints were not included. The broad search combined the disease block (“Carcinoma, Hepatocellular” [MeSH] OR “Liver Neoplasms” [MeSH] OR “hepatocellular carcinoma” [Title/Abstract] OR HCC [Title/Abstract] OR “liver cancer” [Title/Abstract]) with biomarker and modality terms (“Biomarkers, Tumor” [MeSH] OR biomarker*[Title/Abstract] OR “Liquid Biopsy” [MeSH] OR cfDNA [Title/Abstract] OR ctDNA [Title/Abstract] OR methylat*[Title/Abstract] OR fragmentom*[Title/Abstract] OR AFP [Title/Abstract] OR radiomic*[Title/Abstract] OR “Deep Learning” [MeSH] OR “Machine Learning”[MeSH] OR “spatial transcriptom*” [Title/Abstract]) and clinical-use terms (“Early Detection of Cancer” [MeSH] OR surveill*[Title/Abstract] OR diagnos*[Title/Abstract] OR “Immune Checkpoint Inhibitors” [MeSH] OR immunotherap*[Title/Abstract] OR PD-1 [Title/Abstract] OR PD-L1 [Title/Abstract] OR response [Title/Abstract] OR resistance [Title/Abstract] OR neoadjuvant [Title/Abstract] OR perioperative [Title/Abstract] OR “conversion therapy” [Title/Abstract] OR recurrence [Title/Abstract] OR “minimal residual disease” [Title/Abstract]). The complete reproducible string is provided in Supplementary Table S1. The study selection process is detailed in the PRISMA flow diagram (Supplementary Figure S1).

### Biological basis and unmet clinical needs in HCC immunotherapy

The introduction of ICIs has reshaped systemic treatment for advanced HCC. Regimens targeting PD-1 or PD-L1, often combined with anti-angiogenic therapy or other immune agents, have become central components of treatment (Rimassa et al., 2023). However, some patients achieve durable tumor control, whereas others experience primary resistance, early progression or immune-related toxicity without meaningful benefit (Zhu H. et al., 2024). This variability explains the strong demand for clinically reliable predictive biomarkers.

The determinants of ICI response span tumor-intrinsic genomic alterations, antigen presentation, adaptive and innate immune-cell states, angiogenic signaling, WNT/β-catenin activation, liver-disease etiology and the gut–liver immune axis. Despite intensive investigation, no single marker has shown the robustness, reproducibility and treatment specificity required for routine selection of patients across biologically diverse HCC populations.

PD-L1 expression, tumor mutational burden, WNT/β-catenin activation, tumor-infiltrating lymphocytes, circulating markers and imaging-derived signatures have received particular attention. PD-L1 expression has shown inconsistent associations with response and is not sufficiently reproducible for routine patient selection in HCC (Greten et al., 2023). Activating CTNNB1 mutations and WNT/β-catenin signaling are associated with an immune-excluded phenotype in several molecular and clinical cohorts, including impaired dendritic-cell recruitment and reduced CD8^+^ T-cell infiltration (Pinyol et al., 2019). However, CTNNB1 status is not an absolute marker of resistance, and prospective evidence is insufficient to exclude patients from ICI therapy on this basis alone. Conversely, interferon-γ signaling, activated cytotoxic T cells and other immune-inflamed features have been associated with greater ICI sensitivity, whereas immunosuppressive macrophages, regulatory T cells and myeloid-derived suppressor cells have been linked to poorer outcomes (Sung, 2022; Dai et al., 2025). These observations support a model in which response emerges from interactions between tumor-intrinsic programs and the surrounding immune microenvironment rather than from a single molecular feature.

HCC differs from many other solid tumors because it develops within an immunologically specialized organ exposed to chronic inflammation, portal microbial products, metabolic stress and cirrhosis-associated immune dysfunction. Its microenvironment is shaped not only by malignant hepatocytes but also by fibrotic stroma, liver-resident macrophages, endothelial cells, hepatic stellate cells and persistent viral or metabolic injury. The biological context in which an HCC arises can therefore influence both biomarker measurements and treatment response.

In fibrotic and cirrhotic livers, reciprocal signaling among stromal, endothelial and immune populations can establish an immunosuppressive niche that restricts T-cell infiltration and function. Recent spatial transcriptomic studies have implicated DLL4–NOTCH3 signaling between capillary endothelial cells and matrix-producing cancer-associated fibroblasts in immune exclusion (Ji et al., 2026). Fibrosis-associated GOLM1–EGFR/STAT3 signaling may promote PD-L1 expression (Ke et al., 2021), while hypoxia-associated stromal remodeling enriched for SPP1–CD44 communication has been described in scirrhous HCC (Chen et al., 2024). These findings are biologically informative, but most remain discovery-stage or mechanistic observations rather than clinically validated selection biomarkers.

Single-cell profiling of HBV-associated HCC treated with anti-PD-1 plus lenvatinib identified enrichment of GZMK^+^ effector-memory and progenitor-exhausted T-cell states in responders, whereas KIR^+^ CD8^+^ T cells and regulatory T cells were more prominent in non-responders (Gu et al., 2025). PLAUR^+^ neutrophils have been shown experimentally to drive anti-PD-1 resistance by suppressing cytotoxic T-cell activity (Liu S. et al., 2025). Separately, ETV5 has been implicated in HCC progression and recruitment of polymorphonuclear myeloid-derived suppressor cells, supporting an immunosuppressive milieu; that study did not, however, establish ETV5 as a treatment-specific biomarker of ICI resistance (Zhang Z. et al., 2025). Collectively, these studies suggest that cellular state and spatial organization may be more informative than immune-cell abundance alone. Their translation will require reproducible assays, independent validation and evidence that the resulting classifications improve treatment decisions.

The spatial and temporal heterogeneity of HCC is therefore unlikely to be captured by a single biopsy or biomarker. Clinically useful strategies will need to combine mechanistic information from tissue with noninvasive measurements that can sample the whole disease burden and be repeated over time. This need provides the rationale for integrating serum markers, liquid biopsy and AI-assisted imaging in the sections that follow.

### Evidence categories and intended clinical use

Biomarker terminology in this review follows intended clinical use. Diagnostic or surveillance biomarkers identify or rule out disease in a defined at-risk population; prognostic biomarkers are associated with outcomes regardless of treatment choice; predictive biomarkers identify differential benefit from one treatment rather than another and therefore require comparative evidence of a biomarker-by-treatment interaction; and pharmacodynamic or monitoring biomarkers change after treatment and may reflect biological response, tumor burden, or emerging resistance. This framework is applied across serum, cfDNA/ctDNA, tissue, spatial, and imaging biomarkers (Table 1). At present, no biomarker discussed in this review has sufficiently validated treatment-interaction evidence to guide routine selection among ICI regimens in HCC. Accordingly, an association with response within a single treated cohort is not considered sufficient to establish treatment-specific prediction, and terms such as “response-associated” and “monitoring candidate” are used when a formal treatment interaction has not been demonstrated.

**TABLE 1:** Classification of representative HCC biomarkers by intended use and evidence category.

Biomarker/modality	Intended use	Evidence category	Treatment interaction	Current interpretation	Clinical status
AFP with ultrasound	HCC surveillance	Diagnostic/surveillance	Not applicable	Accessible but limited sensitivity; affected by hepatitis and tumor biology	Guideline-supported adjunct to imaging
AFP-L3, DCP/PIVKA-II, composite scores	Detection/risk stratification	Diagnostic or prognostic	Not demonstrated	May add information in selected populations; thresholds vary	Region- and context-dependent
Baseline AFP, NLR, CRP, CRAFITY, ALBI	Pretreatment risk stratification	Predominantly prognostic	No	Reflect tumor burden, inflammation or hepatic reserve	Not validated for ICI-regimen selection
AFP kinetics	On-treatment assessment	Monitoring/pharmacodynamic	No	Associated with radiological response and outcome; definitions vary	Investigational adjunct
cfDNA methylation/fragmentomics	Early detection/tissue-of-origin	Diagnostic/surveillance	Not applicable	High discrimination in selected cohorts; PPV requires surveillance validation	Investigational
Baseline ctDNA/cfDNA burden	Risk stratification	Prognostic	No	Associated with burden and survival	Investigational
Serial ctDNA/VAF	Treatment response/MRD	Monitoring/pharmacodynamic	No	Dynamic molecular burden; no validated action threshold	Investigational
PD-L1, TILs, TLSs	Immune phenotyping	Response-associated/prognostic	Not validated	Biologically plausible but spatially heterogeneous	Not used for routine ICI selection
CTNNB1/WNT activation	Resistance biology	Response-associated/prognostic	Not validated	Candidate immune-exclusion feature; not an absolute resistance marker	Should not exclude ICI
Radiomics/deep learning	Response or outcome estimation	Exploratory prognostic/monitoring	Usually not tested	Predominantly retrospective with limited locked external validation	Research use
Validated predictive marker	Choice between treatments	Predictive	Required	Must show marker-by-treatment interaction in comparative data	None established for routine ICI-regimen selection

### Tissue-based biomarkers and the tumor microenvironment

Tissue specimens provide direct information on malignant cell identity, tumor differentiation, immune infiltration and stromal organization, thereby offering mechanistic insights into HCC diagnosis and why individual patients respond differently to immune checkpoint inhibitors (ICIs). However, unlike several immunogenic cancers, clinically validated tissue biomarkers for immunotherapy selection in HCC remain limited, reflecting the complex interplay between tumor-intrinsic alterations, chronic liver disease-associated immune dysfunction and spatial heterogeneity within the tumor microenvironment (TME).

### Histopathological and molecular biomarkers for HCC characterization

Tissue biopsy remains essential for defining tumor lineage, molecular characteristics and immune microenvironment in hepatocellular carcinoma (HCC). Conventional hepatocellular differentiation markers, including hepatocyte paraffin 1 (HepPar-1), arginase-1, glypican-3 (GPC-3) and alpha-fetoprotein (AFP), are widely used to support the diagnosis of HCC, particularly in poorly differentiated tumors where morphological assessment alone may be insufficient (Wang et al., 2020). Among these markers, arginase-1 and HepPar-1 demonstrate relatively high specificity for hepatocellular differentiation, whereas GPC-3 and AFP are frequently expressed in malignant hepatocytes and may assist in identifying early or poorly differentiated HCC (Shirakawa et al., 2009; Radwan and Ahmed, 2012; Sideras et al., 2015). Conversely, cytokeratin 7 (CK7) and cytokeratin 19 (CK19) are primarily markers of biliary epithelial differentiation and are commonly used to distinguish intrahepatic cholangiocarcinoma (CCA) from HCC (Z et al., 2023). Nevertheless, a subset of HCCs, particularly those with progenitor-like or stem cell features, may express CK7 or CK19, which has been associated with aggressive biological behavior and poorer prognosis (Figure 1).

**FIGURE 1:**
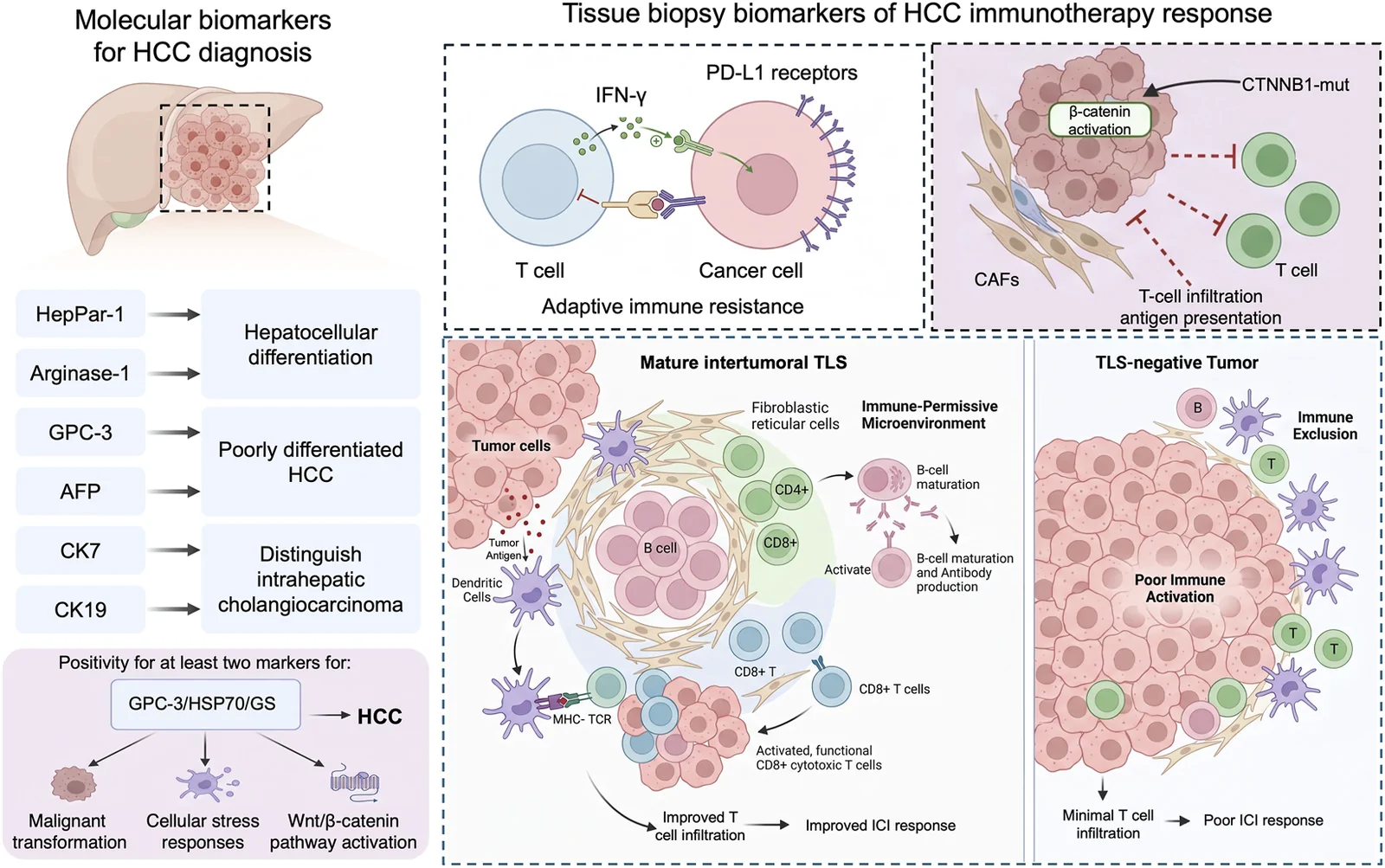
Diagnostic tissue biomarkers for hepatocellular carcinoma and predictive indicators of immune response. The left panel summarizes representative tissue biomarkers used for pathological diagnosis of HCC, whereas the right panel illustrates tissue-derived biomarkers associated with immune checkpoint inhibitor response, including PD-L1 expression, CTNNB1/β-catenin activation and tertiary lymphoid structures (TLSs). AFP, alpha-fetoprotein; CAFs, cancer-associated fibroblasts; CK7/CK19, cytokeratin 7/19; GPC-3, glypican-3; GS, glutamine synthetase; HCC, hepatocellular carcinoma; HepPar-1, hepatocyte paraffin-1; HSP70, heat shock protein 70; ICI, immune checkpoint inhibitor; IFN-γ, interferon-gamma; MHC, major histocompatibility complex; TCR, T-cell receptor; TLS, tertiary lymphoid structure.

Beyond lineage identification, combined immunohistochemical panels have improved the diagnostic accuracy of early HCC detection. A classic example is the combination of GPC-3, heat shock protein 70 (HSP70) and glutamine synthetase (GS), in which positivity for at least two markers supports the diagnosis of HCC in early lesions (Tremosini et al., 2012; Di Tommaso et al., 2009; Di Tommaso et al., 2007). These markers reflect distinct biological processes, including malignant transformation, cellular stress responses and Wnt/β-catenin pathway activation. Although primarily developed for pathological diagnosis, such molecular phenotyping may provide additional information regarding tumor biology relevant to treatment response.

### Tissue biopsy biomarkers of HCC immunotherapy response

Recent studies have expanded the role of tissue biomarkers beyond diagnosis toward immune classification. The advent of spatial transcriptomics, multiplex immunohistochemistry (mIHC), imaging mass cytometry and digital pathology has enabled simultaneous characterization of tumor cells, immune populations and stromal components within their spatial context (Kim EN. et al., 2023; Rigamonti et al., 2024; Semba and Ishimoto, 2024; Koh et al., 2020).

Programmed death-ligand 1 (PD-L1) expression remains the most extensively investigated tissue biomarker for predicting response to PD-1/PD-L1 blockade. PD-L1 upregulation reflects adaptive immune resistance mediated by inflammatory cytokines, particularly interferon-γ produced by activated T cells (Figure 1) (Li et al., 2022; Yi et al., 2018). However, unlike non-small-cell lung cancer and other malignancies in which PD-L1 immunohistochemistry has established clinical utility, its predictive value in HCC remains inconsistent (Hirsch et al., 2017). Several clinical studies have reported associations between PD-L1 expression and immune checkpoint inhibitor response, but the lack of standardized scoring systems, intratumoral heterogeneity and dynamic changes during treatment have limited its application as a standalone biomarker (de Souza et al., 2026; Yoon et al., 2022).

Tumor mutational burden (TMB) and microsatellite instability (MSI) represent additional tissue-based genomic biomarkers reflecting tumor immunogenicity. High TMB can increase the generation of neoantigens and enhance immune recognition, thereby predicting ICI sensitivity in several cancer types (Sun et al., 2025; Palmeri et al., 2022). However, HCC generally exhibits a relatively low mutation burden, and TMB-high tumors are uncommon (Sun et al., 2025; Miura et al., 2025; Woller et al., 2021). Similarly, MSI-high status is rare in HCC compared with colorectal and endometrial cancers (Eso et al., 2020; Bonneville et al., 2017). Consequently, although these genomic features provide biological rationale for immunotherapy response, their clinical utility in HCC remains limited.

Recent genomic studies have highlighted that specific molecular subclasses of HCC may influence immune responsiveness. For example, activation of Wnt/β-catenin signaling, particularly CTNNB1 alterations, has been associated with immune exclusion and reduced response to ICIs (Montironi et al., 2023; Dantzer et al., 2024). Integrative genomic and transcriptomic analyses have suggested that HCC tumors with β-catenin activation exhibit reduced T-cell infiltration and impaired antigen presentation, providing a potential explanation for primary resistance to immunotherapy (Figure 1) (Cai et al., 2024; Ruiz de Galarreta et al., 2019). These findings emphasize that tumor-intrinsic alterations may regulate immune accessibility rather than simply determine tumor antigenicity.

However, tumor-intrinsic alterations alone cannot fully explain the heterogeneity of ICI response in HCC, as immune recognition is also determined by the composition and spatial organization of the tumor microenvironment (Zhang et al., 2023). Increasing evidence indicates that the spatial organization of immune cells within the TME is a critical determinant of immunotherapy efficacy (Feng et al., 2024; Liu et al., 2023). HCC develops in a unique immunological environment characterized by chronic inflammation, fibrosis, cirrhosis-associated immune dysfunction and continuous exposure to gut-derived antigens (Liu et al., 2022; Chen C. et al., 2023; Fan et al., 2024; Yashaswini et al., 2024; Liu Y. et al., 2025). This context generates a highly heterogeneous immune landscape composed of cytotoxic lymphocytes, regulatory T cells, tumor-associated macrophages, cancer-associated fibroblasts and endothelial cells (Tufail et al., 2025; Seyhan et al., 2025).

Tumor-infiltrating lymphocytes (TILs), particularly CD8^+^ T cells, represent one of the most extensively studied immune biomarkers (Luo et al., 2025). High densities of intratumoral CD8^+^ T cells correlate with improved response to ICIs (Lassoued et al., 2025; Lanickova et al., 2025). However, in HCC, the predictive value of TIL abundance alone remains insufficient because immune cells may exhibit distinct spatial distributions and functional states. Recent single-cell RNA sequencing studies have demonstrated substantial heterogeneity among T-cell populations in HCC, including exhausted CD8^+^ T cells expressing inhibitory receptors such as PD-1, TIM-3 and LAG-3, as well as dysfunctional immune states associated with chronic liver injury (Tang et al., 2023; Zhang et al., 2019; Sun et al., 2021; Iñarrairaegui et al., 2018; Chen S. et al., 2023). Therefore, assessment of immune cell composition without considering spatial localization and functional phenotype may underestimate the complexity of antitumor immunity.

Recent advances in spatial profiling technologies have provided new insights into immune organization associated with treatment response. Spatial transcriptomic analysis of HCC samples revealed distinct immune niches characterized by differential interactions between malignant cells, myeloid populations and lymphocytes. These spatial immune states were associated with immune activation patterns and may help explain heterogeneous responses to immunotherapy (Liu et al., 2023; Prawira et al., 2025; Wang et al., 2022; Ye et al., 2024; Li Y. et al., 2025). Similarly, multiplex imaging studies have shown that the spatial proximity between CD8^+^ T cells and tumor cells, rather than overall lymphocyte density alone, may better reflect effective immune surveillance.

Tertiary lymphoid structures (TLSs) have recently emerged as promising tissue biomarkers of antitumor immunity (Lin et al., 2025; Meylan et al., 2022). TLSs are organized lymphoid aggregates containing B cells, T cells, dendritic cells and specialized stromal networks that facilitate local antigen presentation and immune activation (Chen et al., 2026; Cho et al., 2026). Across multiple tumor types, including melanoma, lung cancer and renal cell carcinoma, the presence of mature TLSs has been associated with improved response to ICIs (Tang et al., 2024; Pham et al., 2026; Wu et al., 2022; Hernández-Verdin et al., 2026). In HCC, investigations using spatial transcriptomics and multiplex immunohistochemistry have identified TLS-associated immune niches enriched for activated B cells, antigen-presenting cells and cytotoxic lymphocytes (Zeng et al., 2026; Li et al., 2026). These findings suggest that TLS formation may represent an indicator of a pre-existing immune-permissive microenvironment. Importantly, TLS-based assessment may provide complementary information to widely investigated biomarkers such as PD-L1 expression and TMB. Whereas PD-L1 expression reflects checkpoint-mediated immune suppression and TMB indicates the potential for neoantigen generation, TLSs capture the spatial organization and functional maturation of local adaptive immune responses within the tumor microenvironment (Figure 1).

Nevertheless, tumor heterogeneity, limited tissue biopsy material and the inability to repeatedly sample tumors during therapy restrict their longitudinal application. These limitations are particularly relevant in advanced HCC, where treatment decisions often rely on noninvasive monitoring. Future biomarker strategies will therefore require integration of tissue-based information with circulating and imaging-derived biomarkers.

### Blood-based biomarkers in HCC

Blood-based biomarkers provide a minimally invasive approach for assessing tumor biology, disease evolution and treatment response throughout the HCC care continuum. Unlike tissue-based assays, which are constrained by tumor heterogeneity and limited tissue availability, circulating biomarkers can be repeatedly measured to enable longitudinal monitoring during surveillance, diagnosis, systemic therapy and post-treatment follow-up. However, the sensitivity of conventional serum biomarkers is insufficient for detecting early-stage HCC, and their ability to capture tumor heterogeneity or predict immunotherapy response remains limited. These limitations have stimulated the development of next-generation blood-based biomarkers, including circulating tumor DNA (ctDNA), cell-free DNA (cfDNA) methylation patterns, cfDNA fragmentomic signatures and other liquid biopsy approaches (Cottrell et al., 2025). By capturing tumor-derived molecular alterations and dynamic changes during treatment, these emerging biomarkers may complement conventional serological markers and provide additional biological information for precision HCC management.

### Conventional serum biomarkers for HCC surveillance and early detection

Alpha-fetoprotein (AFP) is the most widely used serum biomarker in HCC surveillance. At the commonly used threshold of 20 ng/mL, AFP has a sensitivity of approximately 60% and a specificity of approximately 90%, although performance varies with disease stage, etiology and the comparison population (Singal et al., 2023; Gambarin-Gelwan et al., 2000; Hanif et al., 2022). AFP may be elevated in active hepatitis or cirrhosis, and many early HCCs are AFP-negative; it should therefore complement, rather than replace, imaging-based surveillance (Tzartzeva et al., 2018). The Lens culinaris agglutinin-reactive AFP fraction (AFP-L3%) and des-γ-carboxy prothrombin (DCP; also known as PIVKA-II) can add information in selected patients, including some with normal total AFP (Feng et al., 2021; Norman et al., 2023; Kim DY. et al., 2023). Neither marker has adequate sensitivity for standalone surveillance. Composite approaches can improve risk stratification, but their incremental clinical benefit, thresholds and availability vary across populations and health systems. Other conventional serum biomarkers that have been investigated for HCC detection are summarized in Table 1.

### Conventional serum biomarkers in HCC immunotherapy

Serum biomarkers are inexpensive and readily repeatable during ICI-based treatment, but their clinical application requires careful definition of intended use. Most available markers do not predict differential benefit from one regimen before treatment. Instead, baseline values usually provide prognostic information, while longitudinal changes may serve as response-monitoring markers.

AFP is the most extensively studied serum marker in this setting. Baseline AFP largely reflects tumor burden and disease severity, whereas early kinetics may add information about treatment response. A meta-analysis of 23 studies involving 2,860 patients found that an AFP decline after ICI treatment was associated with improved overall survival (hazard ratio [HR], 0.40) and progression-free survival (HR, 0.39); rising AFP was associated with poorer outcomes (Zeng et al., 2025). Most component studies were retrospective, and AFP response definitions and sampling windows varied. In a *post hoc* analysis of IMbrave150 among patients with baseline AFP above 20 ng/mL, a decrease of at least 75% at week six identified objective responders, while an increase of no more than 10% identified patients with disease control (Zhu et al., 2022). These thresholds were derived for different endpoints and should not be merged into a single definition of ‘AFP response’.

Inflammation-related indices provide additional, predominantly prognostic, information. A high baseline neutrophil-to-lymphocyte ratio (NLR) has been associated with poorer outcomes in retrospective cohorts and may reflect systemic inflammation and immunosuppressive host environment (Zhang et al., 2026; Zhou et al., 2025). Composite scores can combine tumor-related and host factors. The CRAFITY score, which uses C-reactive protein and AFP, stratified radiological response, progression-free survival and overall survival in patients receiving PD-(L)1 inhibitors (Scheiner et al., 2022). Its simplicity is attractive, although prospective evaluation is needed before it can guide treatment selection.

Hepatic reserve must be considered separately from antitumor sensitivity because liver dysfunction influences eligibility, treatment tolerance and competing mortality. A meta-analysis of 41 studies including 4,483 patients found that baseline liver function was strongly associated with outcomes after ICI treatment. The albumin–bilirubin (ALBI) grade, calculated from objective laboratory measurements, showed consistent prognostic associations with overall and progression-free survival (Tian et al., 2023). ALBI should therefore be viewed as a liver-function and prognostic measure, not as a tumor-specific predictor of ICI efficacy.

Taken together, dynamic AFP is the most extensively studied serum-based monitoring candidate, whereas NLR, C-reactive protein, CRAFITY and ALBI provide complementary, predominantly prognostic information about systemic inflammation and hepatic reserve. Heterogeneous thresholds and retrospective designs limit their standardization. None currently fulfills the evidentiary requirements for selecting one ICI regimen over another.

### cfDNA methylation and fragmentomics for early HCC detection

Cell-free DNA comprises short DNA fragments released into the circulation from malignant and non-malignant cells through apoptosis, necrosis and other cellular processes (Wha et al., 2025; K et al., 2024). ctDNA is the tumor-derived fraction of cfDNA and can carry genetic, epigenetic and fragmentation signatures that reflect tumor biology. Total cfDNA concentration is not tumor-specific and may also rise with inflammation, tissue injury or impaired clearance. Advances in sequencing and computational analysis have extended liquid biopsy beyond mutation detection to methylation profiling, fragmentomics and tissue-of-origin inference (Conway et al., 2024; Esfahani et al., 2022) (Figure 2).

**FIGURE 2:**
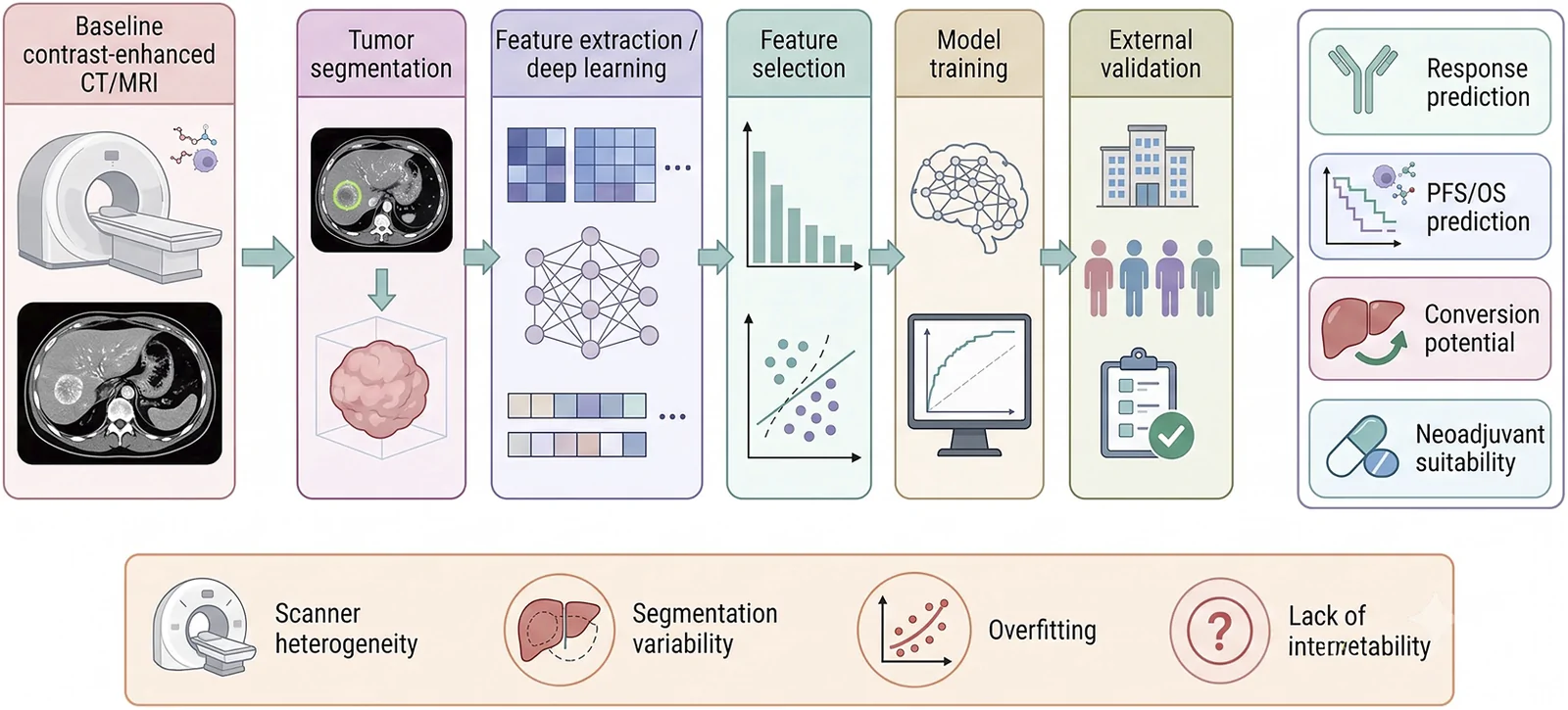
Schematic overview of cfDNA methylation and fragmentomics for early detection of hepatocellular carcinoma. Aberrant methylation and fragmentation features are integrated into a combined model to improve sensitivity for early-stage HCC while maintaining specificity against benign liver diseases and facilitating selection for curative treatment. CNAs, copy number alterations.

Fragmentomics examines properties that are not captured by sequence variants alone, including fragment-size distributions, preferred end coordinates, end motifs, nucleosome footprints and jagged ends. These features are influenced by chromatin organization, nuclease activity, cell death and tissue-specific nucleosome positioning (Zh et al., 2022; Helzer et al., 2025). Malignant and non-malignant liver disease can therefore generate distinct, but potentially overlapping, genome-wide fragmentation patterns.

Several studies have shown the diagnostic potential of these signals. In a multicenter study of 724 individuals with HCC or at average or high risk for HCC, a machine-learning model integrating fragmentomic features detected HCC with 88% sensitivity at 98% specificity in the average-risk cohort and 85% sensitivity at 80% specificity in the high-risk cohort (Foda et al., 2023). In a separate low-coverage whole-genome sequencing study, a stacked ensemble detected stage I and stage II primary liver cancers with sensitivities of 95.9% and 97.9%, respectively (Zhang et al., 2022).

cfDNA methylation is one of the most extensively investigated epigenetic approaches for early HCC detection. Aberrant methylation arises during hepatocarcinogenesis and can affect many genomic loci, providing a broader signal space than a small panel of somatic mutations. Methylation patterns can also contribute tissue-of-origin information. However, chronic inflammation, fibrosis, age and technical platform differences can influence circulating methylation profiles and must be addressed during validation (Zhu Q. et al., 2024). In a multicenter study, the LC-HMC model, based on methylation markers in RNF135, CHFR and PAX5, achieved an area under the receiver operating characteristic curve (AUC) of 0.9714 (95% confidence interval [CI], 0.9603–0.9824) for distinguishing liver cancer from benign liver disease and an AUC of 0.9584 (95% CI, 0.9367–0.9801) for distinguishing liver cancer from other malignancies (Zha et al., 2026).

The potential value of combining orthogonal cfDNA features is illustrated by HIFI, which integrates 5-hydroxymethylcytosine signatures, fragment end motifs, fragmentation patterns and nucleosome footprints. In an independent test cohort, HIFI distinguished HCC from cirrhosis with an AUC of 0.996, 95.4% sensitivity and 97.8% specificity, outperforming AFP in that study (Chen et al., 2021). Collectively, these studies illustrate the rapid evolution of cfDNA-based approaches, from single-marker methylation assays to integrated classifiers incorporating genome-wide methylation profiles, fragmentation patterns and other molecular features. Representative cfDNA-based assays for early HCC detection, together with their reported performance and key limitations, are summarized (Table 2).

**TABLE 2:** Representative serum and cfDNA-based approaches for HCC surveillance or early detection.

Assay/marker	Design/population	n	Comparator	Performance	Threshold/platform	External testing	Main limitation
AFP	Surveillance studies/meta-analyses	Varied	Chronic liver disease/no HCC	At 20 ng/mL: sensitivity ∼60%; specificity ∼90%	20 ng/mL; serum immunoassay	Broad clinical experience	AFP-negative early tumors; inflammation-related elevation
Multifeature fragmentomics (Esfahani et al., 2022)	Multicenter; average- and high-risk cohorts	724	Average- or high-risk individuals without HCC	Average risk: 88% sensitivity at 98% specificity; high risk: 85% sensitivity at 80% specificity	Machine-learning fragmentomic classifier; detailed threshold NR	Multicenter cohort	Lower specificity in high-risk population; surveillance PPV not established
Stacked low-pass WGS model (Zh et al., 2022)	Case-control/selected primary liver cancer cohorts	NR	Non-cancer controls	Stage I sensitivity 95.9%; stage II sensitivity 97.9%	Low-coverage WGS ensemble; threshold NR	Independent test reported	Selected spectrum; liver-cancer rather than exclusively HCC endpoint
LC-HMC methylation model (Foda et al., 2023)	Multicenter liver-cancer study	NR	Benign liver disease/other malignancies	AUC 0.9714 (95% CI 0.9603-0.9824) vs benign liver disease; AUC 0.9584 (95% CI 0.9367-0.9801) vs other malignancies	RNF135, CHFR and PAX5 methylation; threshold NR	Multicenter validation	Platform and threshold transferability require confirmation
HIFI multi-omics cfDNA (Zhang et al., 2022)	Independent test cohort; HCC vs cirrhosis	NR	Cirrhosis	AUC 0.996; sensitivity 95.4%; specificity 97.8%	5hmC, end motifs, fragmentation and nucleosome footprints; threshold NR	Independent test cohort	Enriched comparison; prospective PPV and recall burden unknown

Important barriers remain before cfDNA assays can be incorporated into routine surveillance. Many studies are retrospective or case-control in design, increasing the risk of spectrum bias and inflated performance estimates. Early-detection tests should be evaluated in clinically representative cohorts containing cirrhosis, chronic hepatitis, dysplastic nodules and benign liver lesions, rather than healthy controls alone. Because HCC prevalence is much lower in surveillance cohorts than in case-control datasets, even high sensitivity and specificity may yield a modest positive predictive value. Assay platforms, sequencing depth, bioinformatic pipelines and thresholds also vary, limiting direct comparison and reproducibility. Accordingly, surveillance assays should be evaluated using positive predictive value, false-positive recall burden, interval-cancer detection and downstream diagnostic consequences at clinically relevant prevalence, rather than AUC alone.

Important barriers remain before cfDNA assays can be incorporated into routine surveillance. Many studies are retrospective or case-control in design, increasing the risk of spectrum bias and inflated performance estimates. Early-detection tests should be evaluated in clinically representative cohorts containing cirrhosis, chronic hepatitis, dysplastic nodules and benign liver lesions, rather than healthy controls alone. Because HCC prevalence is much lower in surveillance cohorts than in case-control datasets, even high sensitivity and specificity may yield a modest positive predictive value. Assay platforms, sequencing depth, bioinformatic pipelines and thresholds also vary, limiting direct comparison and reproducibility.

### cfDNA and ctDNA in HCC immunotherapy

During immunotherapy, cfDNA and ctDNA offer a repeatable means of assessing molecular burden and clonal dynamics. A longitudinal study of 772 plasma samples found that ctDNA mutation detection increased with tumor stage. Baseline ctDNA mutations were associated with recurrence and mortality after percutaneous ablation, while persistent mutations during atezolizumab–bevacizumab treatment were associated with radiological progression (Campani et al., 2024). These results support serial ctDNA as a monitoring and prognostic tool, but they do not establish that a ctDNA result predicts differential benefit from a particular regimen.

Baseline cfDNA abundance has also shown prognostic associations, but total cfDNA is not tumor-specific. In a prospective cohort of 85 patients receiving atezolizumab plus bevacizumab, higher pretreatment cfDNA concentrations were associated with lower response rate and shorter progression-free and overall survival (Matsumae et al., 2022). In another prospective cohort of 130 patients receiving the same regimen, plasma sequencing showed high concordance with tissue genomic profiling when samples were collected close in time, and maximum variant allele frequency was independently associated with survival (Hwang et al., 2025). These findings support quantitative and longitudinal assessment for risk stratification and monitoring, while underscoring the absence of a validated treatment-selection threshold.

### Cytotoxic chemotherapy resistance in HCC

Cytotoxic chemotherapy has a limited and region-dependent role in advanced HCC but remains relevant to fluorouracil-leucovorin-oxaliplatin regimens and to anthracycline- or platinum-containing locoregional treatment. Candidate resistance mechanisms include increased drug efflux through ATP-binding cassette transporters such as ABCB1/MDR1, ABCC1/MRP1 and ABCG2/BCRP; altered drug metabolism; enhanced DNA repair; impaired apoptosis; and activation of PI3K-AKT-mTOR, MAPK-ERK, WNT/β-catenin and NF-κB signaling (Marin et al., 2020; Ladd et al., 2024).

Cancer-stem-cell and progenitor-like states marked by CD133, EpCAM, CD90 or CK19 may combine high drug-efflux capacity with quiescence, DNA-repair competence and resistance to apoptosis. Epithelial-mesenchymal plasticity, hypoxia-HIF-1α signaling, cancer-associated fibroblasts, NRF2-mediated redox adaptation, autophagy, ferroptosis regulation and anti-apoptotic proteins such as BCL-2 and survivin can further reduce sensitivity to fluoropyrimidines, oxaliplatin and anthracyclines. Most supporting evidence is derived from cell lines, animal models or retrospective tumor analyses, and no marker is validated for routine selection of a cytotoxic regimen in HCC.

The reviewer-requested pan-cancer analysis identified CDK2, ID1, DUSP6, c-JUN, PLA2G2A and SKP2 in colorectal and ovarian cancer datasets and evaluated selected candidates in oral cancer samples. These findings are cited as hypothesis-generating cross-cancer evidence rather than as proof of chemoresistance biomarkers in HCC.

### AI-based imaging biomarkers for immunotherapy

Cross-sectional imaging is central to HCC diagnosis, staging, treatment planning and response assessment. Contrast-enhanced CT and MRI depict tumor size, arterial hyperenhancement, washout, capsule appearance, vascular invasion, satellite nodules and extrahepatic spread (Jiang et al., 2026; Kim et al., 2022). Routine interpretation, however, remains predominantly qualitative, and size- or enhancement-based response criteria cannot fully represent the biological heterogeneity that shapes tumor evolution and immunotherapy response.

Radiomics converts CT or MRI into quantitative descriptors of morphology, intensity, texture, margin complexity, intratumoral heterogeneity and peritumoral architecture. These imaging phenotypes can be associated with angiogenesis, fibrosis, hypoxia, immune infiltration and vascular invasion (Rostami et al., 2026; Liu H. et al., 2026). Unlike a biopsy, which samples a limited region, radiomics evaluates the entire visible tumor and its surroundings and can be repeated during treatment. This broader sampling is an advantage, but it does not provide direct molecular measurement and remains sensitive to acquisition and segmentation choices (Zeng et al., 2024; Qi et al., 2024).

Retrospective clinical cohorts provide proof of concept. In 58 patients with advanced HCC, a contrast-enhanced CT radiomics nomogram incorporating tumor embolus and ALBI grade achieved AUCs of 0.894 and 0.883 in training and validation cohorts for anti-PD-1 response (Yuan et al., 2020). Similarly, a separate CT model integrating radiomic features with AFP and NLR achieved AUCs of 0.897 and 0.888 and also stratified progression-free survival (Cui et al., 2023). Beyond CT-based approaches, pretreatment MRI signatures predicted response to lenvatinib plus an anti-PD-1 antibody with AUCs of 0.886 and 0.820 in training and validation cohorts (Xu et al., 2023). More recently, a whole-liver longitudinal CT model developed in 395 patients across three institutions reported AUCs of 0.883, 0.876 and 0.875 in training, internal-validation and external-validation cohorts, respectively (Xu et al., 2025). Notably, an MRI-derived radiomic immunoscore linked imaging to spatial immune profiling and achieved an AUC of 0.753 for tumor-immune phenotype and 0.731 for anti-PD-1 response (Wu et al., 2023).

Radiogenomics can help interrogate the biological basis of imaging signatures. One contrast-enhanced CT study combined a radiomic feature with SPOCD1, KLRB1 and GPR182 expression to construct a tumor-microenvironment-related prognostic model. The model achieved 1-, 2- and 3-year overall-survival AUCs of 0.81, 0.85 and 0.87 in the training set and 0.73, 0.83 and 0.84 in the test set; higher scores were associated with lower immune infiltration (Wang et al., 2023). This example supports biological interpretation and prognostic stratification, but it was not a prospective test of ICI treatment selection.

Multimodal models can combine imaging with clinical or molecular measurements. A multicenter study developed machine-learning radiomic models for outcomes after atezolizumab plus bevacizumab and found that radiomic approaches outperformed established clinical biomarkers in its study cohorts (Vithayathil et al., 2025). Other reports suggest that combining imaging with AFP, liver-function indices or inflammatory markers may improve discrimination. However, incremental performance within a development dataset does not establish clinical utility. Comparisons must use identical external cohorts, prespecified endpoints and clinically relevant reference models. Evidence from radiomics studies of postoperative recurrence cannot be assumed to apply to immunotherapy response.

Deep learning can learn hierarchical representations directly from medical images and thereby reduce reliance on manually engineered features (Fusco et al., 2025; Liu J. et al., 2025). Convolutional and transformer-based architectures may capture complex spatial relationships among tumor phenotype, vascular characteristics, the surrounding liver parenchyma, and imaging correlates of the immune microenvironment (Liu K. et al., 2026; Karabağ et al., 2026). However, their clinical utility remains dependent on rigorous image curation, standardized acquisition protocols, robust segmentation strategies, and external validation. Apparent model performance may be inflated by data leakage, spectrum bias, or institution-specific imaging characteristics, limiting generalizability across clinical settings (Chouvarda et al., 2025; Suleman et al., 2025) (Figure 3).

**FIGURE 3:**
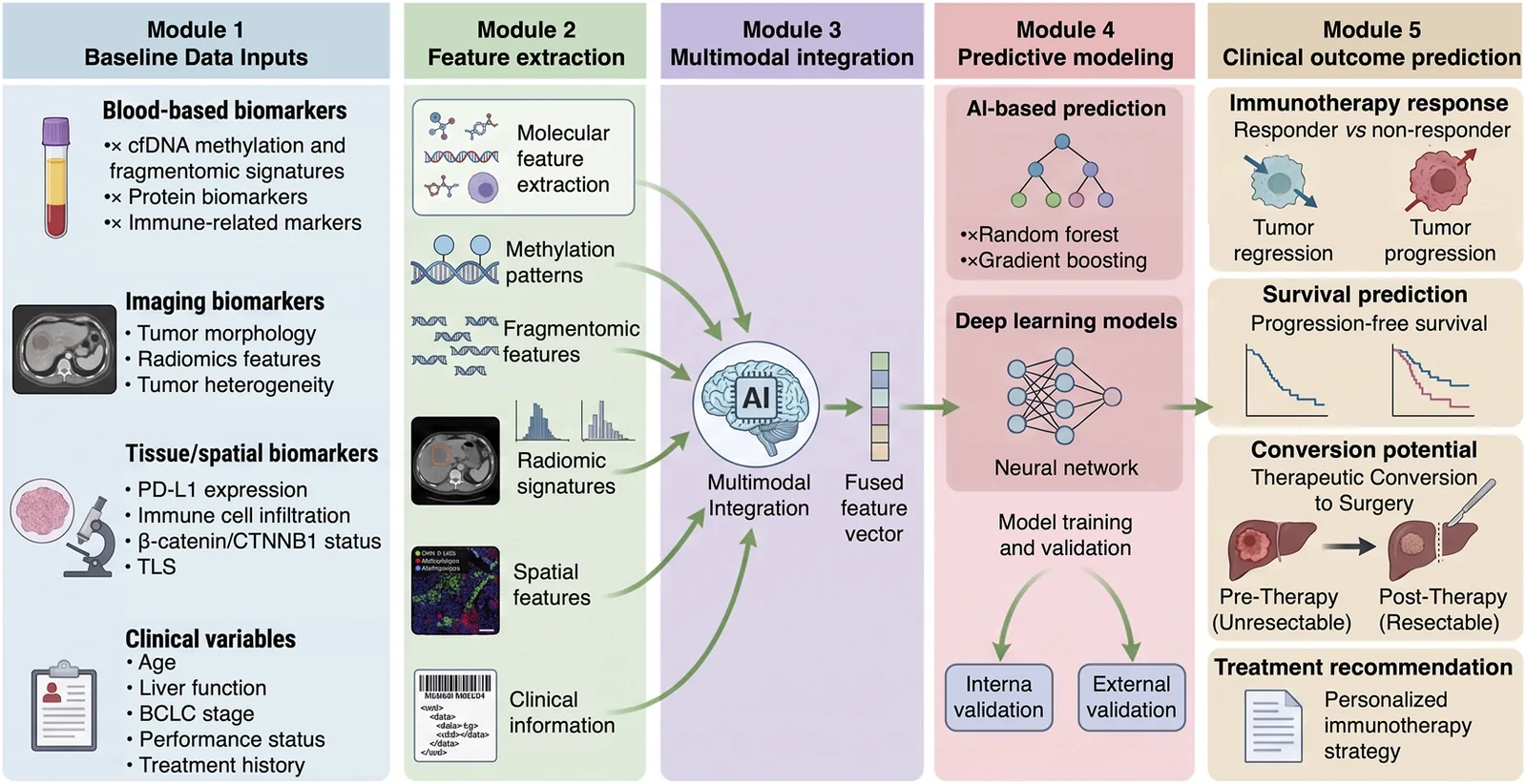
Standard workflow of AI-assisted radiomics for predicting immunotherapy outcomes in HCC. Contrast-enhanced CT and MRI images undergo tumor segmentation, feature extraction, model development and external validation to predict treatment response, survival and neoadjuvant suitability, while highlighting current technical limitations.

Before an AI-based imaging biomarker can be considered clinically transferable, it should be evaluated in representative multicenter cohorts using patient-level and preferably site- or time-separated data partitions; use prespecified endpoints and reference standards; perform preprocessing and feature selection within training folds; report missing-data handling and events relative to model complexity; test robustness across scanners, acquisition protocols and segmentation methods; and evaluate a locked model and threshold in an independent external cohort. Discrimination should be accompanied by calibration, comparison with an established clinical model, decision-curve analysis and, ultimately, prospective clinical-impact evaluation. CLEAR and the Image Biomarker Standardization Initiative support radiomics transparency and reproducibility, while CLAIM 2024, TRIPOD + AI and PROBAST + AI provide complementary standards for AI reporting and risk-of-bias assessment.

### Multimodal assessment in neoadjuvant immunotherapy

Neoadjuvant immunotherapy is under active investigation in HCC but is not established routine care. Upfront resection remains the standard curative approach for suitable patients, although recurrence is common, particularly with large tumor size, microvascular invasion, satellite nodules, poor differentiation and elevated AFP levels (Herrero et al., 2024; Meng et al., 2025). Preoperative immunotherapy may induce tumor regression, expose occult systemic disease and enhance antitumor immunity while tumor antigens remain present (Silihe Kamga and Fiering, 2025). These potential advantages must be balanced against toxicity, surgical delay and progression beyond resectability. Conversion therapy is a distinct strategy aimed at transforming initially unresectable disease into a state suitable for local curative-intent treatment. Accordingly, neoadjuvant and conversion cohorts should not be combined without explicit stratification (Llovet et al., 2024; Cai et al., 2025).

The neoadjuvant setting also provides a valuable translational framework. Pretreatment biopsy, serial plasma sampling, on-treatment imaging and the resection specimen can be analyzed within the same patient. This design enables direct study of radiological and pathological response, immune microenvironment remodeling, clonal evolution and residual viable tumor. It can therefore support biomarker discovery, but small single-arm trials remain vulnerable to selection bias and cannot establish that a biomarker is predictive without an appropriate comparator (Funt and Chapman, 2016; Valenza et al., 2025; Liu N. et al., 2025).

Two challenges are especially important. First, not every patient with technically resectable, high-risk HCC should receive preoperative systemic therapy; unnecessary treatment may cause toxicity or delay potentially curative surgery. Second, conventional imaging can misclassify immune-related changes, necrosis or delayed regression. A biomarker framework should therefore address a defined decision—eligibility, early continuation, surgical timing or postoperative surveillance—rather than combine all endpoints into one model. The term “borderline resectable” is not standardized in HCC and should not be used without protocol-specific criteria. Studies using this term should report anticipated margin status, major vascular or biliary involvement, tumor distribution, future liver remnant, liver function and multidisciplinary surgical assessment. The framework therefore distinguishes high-risk but technically resectable disease from initially unresectable disease rather than treating borderline resectability as a universal category (Zhang Y. et al., 2025; Marron et al., 2022).

Complementary modalities could improve both selection and response assessment. Baseline radiomic features may identify imaging phenotypes associated with sensitivity or resistance, whereas serial imaging can quantify changes in vascularity, necrosis and spatial heterogeneity (Han et al., 2024; Elsherif et al., 2022; Antunes et al., 2020). Changes in ctDNA variant allele fraction, methylation or fragmentomic signals may provide molecular evidence of tumor-burden reduction, persistent disease or emerging resistance (Liu et al., 2025e; Giro et al., 2024; Peng et al., 2024; Li S. et al., 2025). Pretreatment and resection tissue can then anchor these noninvasive measurements to immune and pathological endpoints. Each candidate marker should be analysed according to its intended role and sampling time point (Boxberg et al., 2022; Lee et al., 2024) (Figure 4).

**FIGURE 4:**
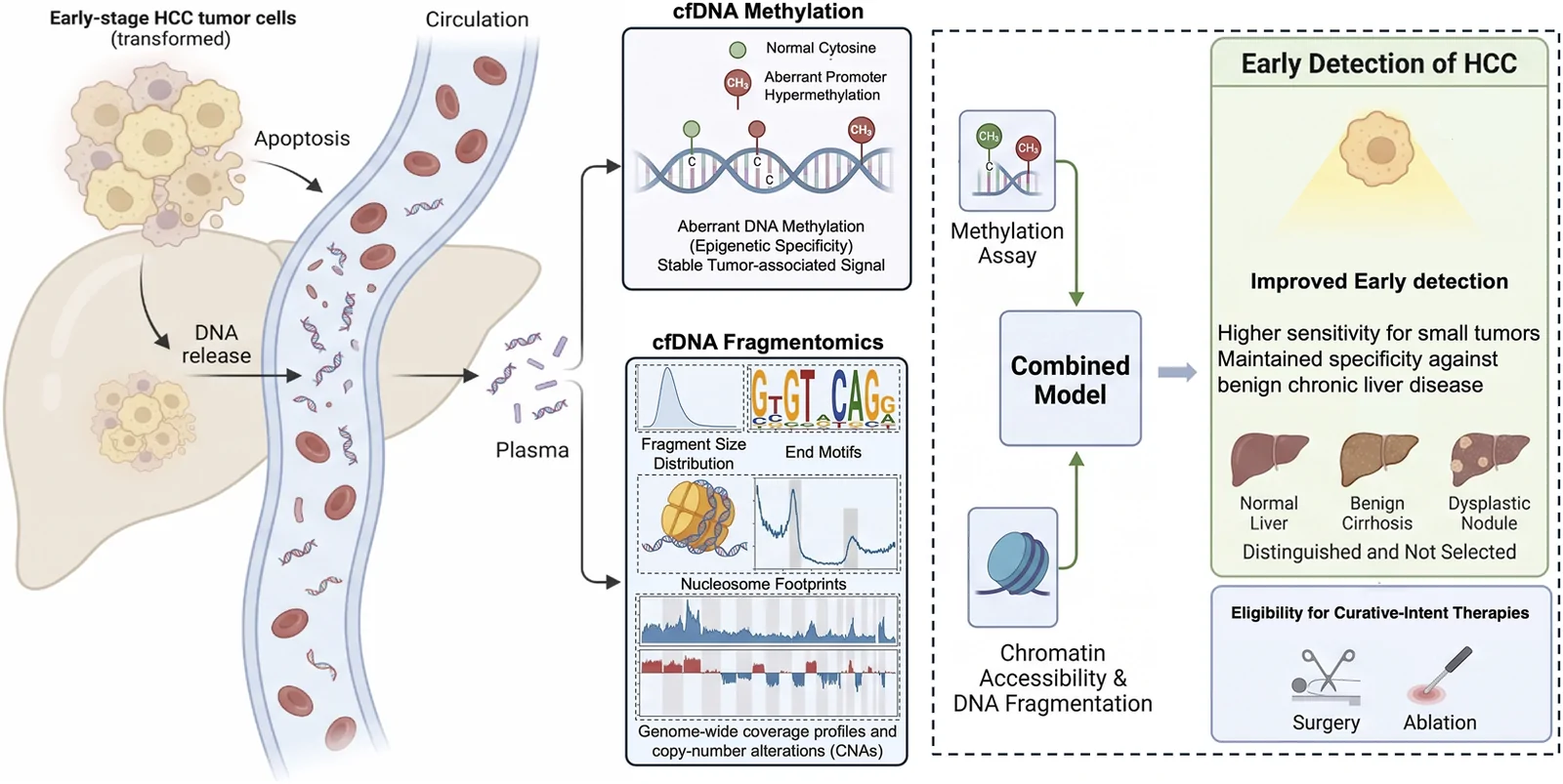
Multimodal biomarker-guided framework for neoadjuvant immunotherapy assessment and perioperative management of hepatocellular carcinoma. Multimodal biomarkers integrate tissue biopsy, liquid biopsy, imaging and clinical variables for baseline risk stratification of HCC patients. High-risk resectable HCC and initially unresectable HCC are distinguished according to different therapeutic goals, including neoadjuvant therapy and conversion therapy. During treatment, serial imaging, plasma biomarkers and tumor immune microenvironment profiling enable dynamic response monitoring and treatment adjustment. Surgical specimens provide pathological endpoints, including major pathological response, pathological complete response and residual viable tumor assessment, for biomarker validation. Postoperative molecular surveillance using circulating biomarkers supports MRD detection and recurrence risk assessment, enabling personalized perioperative management of HCC. MRD, minimal residual disease.

Major pathological response (MPR) and pathological complete response (pCR) are being explored as neoadjuvant endpoints (Liu et al., 2025f; Leidner et al., 2021). Their optimal thresholds and relationships with recurrence-free and overall survival are not yet established in HCC (Rhu et al., 2025; D’Alessio et al., 2024; Yang et al., 2025). Assessment may be complicated by spontaneous necrosis, prior locoregional treatment and heterogeneous residual viability (Hwang et al., 2021). Standardized sampling of the resection specimen and reproducible quantification of residual viable tumor are therefore essential; MPR or pCR should not yet be assumed to be validated surrogate endpoints for long-term benefit (Tian et al., 2024; Wang et al., 2024).

Prospective studies of neoadjuvant immunotherapy should evaluate two clinically distinct populations separately: patients with high-risk but resectable HCC receiving investigational neoadjuvant therapy, and patients with initially unresectable HCC receiving conversion therapy. Their baseline eligibility, treatment goals, selection processes and clinically relevant endpoints differ. Pooling them can obscure whether a biomarker is associated with pathological response, conversion to resectability or general prognosis. Each setting requires predefined biomarker hypotheses, standardized pathological or surgical resectability assessment, serial plasma sampling, harmonized imaging and long-term outcomes. Randomized or otherwise appropriately controlled studies are needed to distinguish treatment-specific prediction value from general prognostic associations.

### Staged implementation roadmap for multimodal biomarker studies

A clinically oriented study should first define the population, intended use, reference standard and action triggered by the biomarker. At baseline, clinical stage, liver function and guideline-concordant CT or MRI constitute the mandatory core; standardized blood sampling can provide exploratory molecular measurements, and tissue should be obtained only when clinically indicated or prospectively embedded in a translational protocol. Early on-treatment sampling at a protocol-defined cycle may evaluate AFP and ctDNA/cfDNA kinetics, but an isolated experimental result should not trigger treatment discontinuation. At the first protocol-defined imaging assessment, RECIST 1.1 or mRECIST, clinical status, hepatic reserve and biomarker trends should be reviewed together.

Preoperative reassessment should separately address pathological response in high-risk resectable neoadjuvant cohorts and conversion to resectability in initially unresectable cohorts. Postoperative studies should link standardized residual viable tumor assessment, MPR or pCR, and fixed-time-point molecular residual disease testing to recurrence-free and overall survival. Model development should progress from retrospective discovery to internal validation, locked temporal or geographic external validation, prospective observational validation and, finally, biomarker-guided impact trials.

Missing-modality handling should be prespecified. Mandatory and optional modalities should be distinguished, availability and reasons for missingness should be reported, and imputation or modality-dropout procedures should be confined to training data. Complete-case and missing-modality sensitivity analyses are needed to determine whether performance depends on an unrealistic subset with complete multimodal data.

## Challenges and future directions

Technical standardization is a prerequisite for clinical use. For serum assays, studies should report platform, units, calibration, limits of detection and quantification, hemolysis handling, sampling time and locked thresholds. For cfDNA/ctDNA, minimum reporting should include blood volume and tube type, time to plasma separation, centrifugation, storage, extraction input and yield, fragment-quality metrics, sequencing depth, error suppression, analytical sensitivity, white-blood-cell or clonal-hematopoiesis controls, batch effects and the prespecified positivity threshold. Methylation and fragmentomic studies should additionally report conversion or enrichment efficiency, CpG or genomic coverage, mapping quality, GC and fragment-size bias and classifier locking. Imaging studies should report scanner and acquisition parameters, contrast timing, reconstruction, voxel resampling, segmentation, interobserver robustness and IBSI-compliant feature definitions. Table 3 summarizes these minimum quality-control and reporting variables by modality (D’Alessio et al., 2024; Yang et al., 2025; Hwang et al., 2021).

**TABLE 3:** Minimum quality-control and reporting standards for multimodal HCC biomarker studies.

Modality	Minimum pre-analytical reporting	Minimum analytical/computational QC	Validation and clinical-utility requirements	Relevant standards
Serum proteins	Sampling time, fasting status if relevant, hemolysis, processing and storage	Platform, units, calibration, LOD/LOQ, batch QC, locked cutoff	Representative population, sensitivity/specificity/PPV, incremental value	STARD where diagnostic
cfDNA/ctDNA	Blood volume, tube, time to plasma, centrifugation, storage and freeze-thaw	Extraction input/yield, fragment profile, depth, error suppression, LOD, CHIP/WBC control, locked positivity rule	Independent testing, serial time points, predefined action threshold and clinical endpoint	STARD/REMARK as applicable
Methylation/fragmentomics	As for cfDNA plus input requirements	Conversion/enrichment efficiency, genomic coverage, mapping quality, GC/size bias, batch handling, locked classifier	Prospective high-risk cohort, PPV, recall burden and interval cancers	Transparent assay and code reporting
Tissue/spatial profiling	Specimen type, ischemia/fixation, sampling regions and tumor content	Antibody/probe identity, scoring, spatial QC, batch correction and reproducibility	Independent cohort, prespecified endpoint, comparison with routine pathology	REMARK and platform-specific guidance
Radiomics	Scanner/vendor, sequence, contrast phase/timing, reconstruction and voxel size	Segmentation method, interobserver stability, preprocessing, IBSI feature definitions and training-fold feature selection	Locked external validation, calibration, DCA and clinical comparator	IBSI; CLEAR
Deep learning/multimodal AI	Data provenance, inclusion, missing modalities and site/time distribution	Patient-level splits, leakage prevention, class imbalance, event-to-complexity, code/model version and robustness testing	Locked site/time external test, calibration, DCA, prospective impact and cost-effectiveness	CLAIM 2024; TRIPOD+AI; PROBAST+AI

Biological and clinical heterogeneity present a second challenge. Chronic inflammation, fibrosis and cirrhosis contribute nontumor cfDNA and alter imaging phenotypes, while HCC etiology and treatment history shape the immune microenvironment (Li J. et al., 2024). Performance in healthy-control case–control studies is therefore unlikely to translate directly to surveillance populations. Validation cohorts should represent the intended clinical setting and include chronic hepatitis, cirrhosis, dysplastic nodules, benign lesions, variable liver function and relevant demographic groups.

Biomarker development must be linked to a specific clinical action. Early detection, pretreatment prognosis, treatment selection, on-treatment monitoring, neoadjuvant pathological response and postoperative minimal residual disease require different samples, reference standards and endpoints. For treatment selection, evidence of a biomarker-by-treatment interaction is more informative than association within a single treatment group. A statistically significant association or high AUC is otherwise insufficient: studies should demonstrate calibration, incremental value beyond current care, a feasible downstream pathway and an acceptable balance of missed disease, false positives, cost and patient burden.

Multimodal integration is attractive because serum, cfDNA, imaging, tissue and clinical variables measure complementary aspects of disease. Greater complexity, however, increases the risk of overfitting, data leakage and loss of interpretability, particularly when the number of candidate features exceeds the number of outcome events. Models should be parsimonious, handle missing modalities explicitly and undergo temporally and geographically independent validation. Decision-curve and cost-effectiveness analyses can help determine whether added complexity produces meaningful clinical benefit.

### Interpretation of discordant multimodal results

Discordance should be resolved according to intended use, analytical validity and temporal relevance rather than by averaging modalities or applying a simple majority rule. The first step is to verify sample quality, timing, treatment exposure and whether the tests answer the same clinical question. Established clinical evidence retains priority: histopathology and guideline-concordant imaging establish diagnosis and anatomy, while clinical status and liver function determine treatment safety. Tissue usually provides a local baseline mechanistic measurement, ctDNA reflects shed systemic molecular burden, and imaging samples whole-disease anatomical and vascular change; disagreement may therefore represent biological compartmentalization rather than analytical failure.

For example, an immune-excluded tissue phenotype may coexist with falling ctDNA and radiological response. If the patient is clinically stable, the dynamic response measures should not be overruled by the baseline tissue phenotype. Conversely, a favorable tissue phenotype should not override confirmed radiological progression or reproducibly rising molecular burden. Serial trends and confirmation with an orthogonal modality are preferred to isolated results, and no investigational biomarker should independently trigger ICI discontinuation, surgery or conversion treatment. The reviewer-requested AREG study in oral squamous cell carcinoma illustrates that tissue and circulating biomarker levels can diverge across biological compartments; it is cited as a cross-cancer example and not as HCC-specific evidence.

Future progress will depend on moving beyond retrospective biomarker discovery toward prospective, decision-oriented clinical validation. This transition will depend on collaborative studies with standardized data collection, prespecified analysis plans, transparent reporting and rigorous external validation. Ultimately, the value of a biomarker lies not simply in its association with clinical outcomes, but in its ability to inform clinical decisions and improve patient outcomes.

## Conclusion

ICI-based therapy has transformed HCC management, yet no biomarker is sufficiently validated to guide routine selection among regimens. The central challenge is biological: tumor-intrinsic alterations interact with the immune and stromal microenvironment of a chronically diseased liver, and both evolve during treatment. The evidentiary challenge is equally important: prognosis, monitoring and treatment-specific prediction require different study designs.

Available modalities answer different questions. Serum markers are accessible measures of tumor burden, inflammation and hepatic reserve; cfDNA methylation and fragmentomics offer early-detection and tissue-of-origin signals; ctDNA can track molecular burden and clonal dynamics; tissue profiling reveals mechanism; and AI-based imaging characterizes whole-tumor and peritumoral heterogeneity (Table 4). The neoadjuvant setting provides an opportunity to connect these measurements with resection pathology and postoperative outcomes (Figure 4).

**TABLE 4:** Methodological and clinical-utility assessment of representative AI-imaging studies in HCC immunotherapy.

Study/model	Design/sites	n	Outcome	Validation	Reported performance	Calibration/DCA/locked threshold	Evidence level/limitation
Yuan et al. 2020, CECT nomogram	Retrospective; single center	58	Anti-PD-1 response	Development plus internal validation	AUC 0.894/0.883	NR/NR/NR	Proof of concept; very small cohort
Cui et al., 2023, CECT + AFP/NLR	Retrospective; setting NR	NR	Response and PFS	Training/validation	AUC 0.897/0.888	NR/NR/NR	Association within treated cohort; no treatment interaction
Xu et al., 2025 pretreatment MRI	Retrospective multicenter	NR	Response to lenvatinib + anti-PD-1	Training/validation	AUC 0.886/0.820	NR/NR/NR	Multicenter but treatment-specific interaction not tested
Xu et al., 2023 longitudinal whole-liver CT	Retrospective; 3 institutions	395	ICI-combination response/prognosis	Training, internal and external validation	AUC 0.883/0.876/0.875	See original report; prospective impact not shown	Strongest external-validation example; still retrospective
Wu et al., 2022 MRI immunoscore	Retrospective translational cohort	NR	Immune phenotype/anti-PD-1 response	Training/test	AUC 0.753/0.731	NR/NR/NR	Biological linkage; moderate discrimination
Wang et al., 2020 radiogenomics	Retrospective	NR	Overall survival	Training/test	1-3 year AUC 0.81-0.87/0.73-0.84	NR/NR/NR	Prognostic, not ICI-treatment selection
Vithayathil et al., 2025, ML radiomics	Multicenter	NR	Outcomes after atezolizumab + bevacizumab	Multicenter validation	Outperformed selected clinical biomarkers in study cohorts	Verify original report; clinical-impact trial absent	Incremental discrimination does not establish net benefit

The distinctive contribution of this review is the alignment of each modality with a defined decision and evidence category, followed by a longitudinal framework that connects surveillance, treatment assessment, preoperative reassessment and postoperative molecular monitoring. The most credible path to precision care is not a universal single marker, but a clinically defined and longitudinal combination of modalities. Analytical standardization, representative prospective cohorts, independent validation and interventional evidence of clinical utility will determine whether these promising tools improve surveillance, immunotherapy management and perioperative care (Figure 5).

**FIGURE 5:**
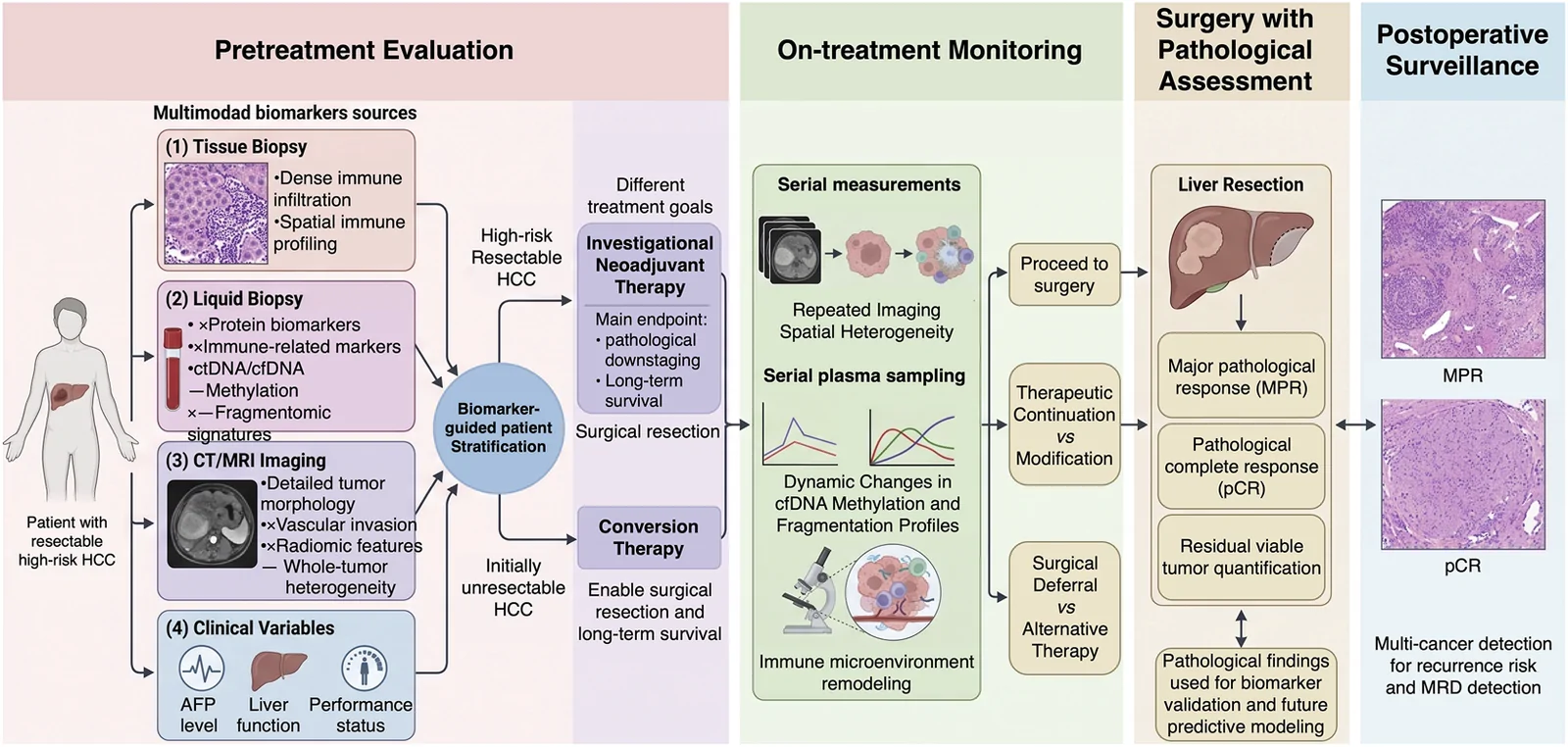
Integrated multimodal model for predicting immunotherapy response in hepatocellular carcinoma. Integration of complementary biomarkers from blood, imaging, tissue/spatial profiling and clinical characteristics enables development of multimodal predictive models for immunotherapy response in HCC. Blood-based biomarkers, including ctDNA, cfDNA methylation, fragmentomic signatures and protein markers, capture systemic tumor-derived signals, whereas imaging and tissue-based biomarkers provide information on tumor heterogeneity, immune microenvironment and spatial features. These heterogeneous data are transformed into integrated feature representations using computational approaches and machine learning/deep learning models. The resulting predictive framework may support clinical decision-making by estimating immunotherapy response, survival outcomes, conversion potential and personalized treatment strategies. AI, artificial intelligence; BCLC, barcelona clinic liver cancer; TLS, tertiary lymphoid structure.

This review has limitations. Its structured narrative design was not preregistered and did not include formal risk-of-bias scoring or meta-analytic synthesis; literature-selection and publication bias are therefore possible. The evidence base is also evolving rapidly, and several cited studies involve selected cohorts or cross-cancer methodology. Established clinical evidence, emerging observational associations and the authors’ proposed multimodal research framework should consequently be interpreted separately. The framework is intended to guide future validation and should not be regarded as a current treatment guideline.
