# Supplementary material for: Multimodal biomarkers for early detection and immunotherapy assessment in hepatocellular carcinoma: integrating liquid biopsy, AI-based imaging and tumor immune profiling
Source: Front Genet. 2026 Oct 5;17:1951687. doi: 10.3389/fgene.2026.1951687 (PMC13678074; doi:10.3389/fgene.2026.1951687)
Supplement: Supplementary file 1 [file DataSheet1.docx]

**Supplemental Figure 1.** PRISMA-style flow diagram for selection of the 61 core HCC-focused studies. PubMed/MEDLINE identified 19,579 records. After the 10-year date restriction, 15,251 records underwent title and abstract screening; 10,819 were considered for full-text retrieval and publication-type eligibility; 2,310 underwent detailed assessment; and 61 met the predefined criteria for the core evidence set. In addition, 124 contextual references were identified through citation screening or targeted methodological and guideline searches and were used for background, standards and explicitly labelled cross-cancer context.

**Identification of studies via databases and registers**

Records removed *before screening*:

Records excluded based on publication date>10 years old(n=4,328)

Records identified from PubMed/MEDLINE(n = 19,579)

**Identification**

Records screened

(n=15,251)

Exclude based on reading abstract(n=4,432)

Excluded by publication type (n = 8,509). Not an original/clinical study or trial,randomized trial, meta-analysis, or review.

Reports sought for retrieval

(n=10,819)

**Screening**

Full texts excluded (n = 2,249).

Non-HCC disease focus (n = 2,047)Local-procedure focus withoutbiomarker relevance (n = 202)

Full texts assessed for relevance and eligibility

(n = 2,310)

Core HCC-focused studies prioritizedfor detailed synthesis (n = 61)

**Included**
